# Murine Dendritic Cells Transcriptional Modulation upon *Paracoccidioides brasiliensis* Infection

**DOI:** 10.1371/journal.pntd.0001459

**Published:** 2012-01-03

**Authors:** Aldo H. Tavares, Lorena S. Derengowski, Karen S. Ferreira, Simoneide S. Silva, Cláudia Macedo, Anamélia L. Bocca, Geraldo A. Passos, Sandro R. Almeida, Ildinete Silva-Pereira

**Affiliations:** 1 Faculdade de Ceilândia, Universidade de Brasília, Brasília, Brasil; 2 Departamento de Biologia Celular, Universidade de Brasília, Brasília, Brasil; 3 Departamento de Ciências Biológicas, Universidade Federal de São Paulo, São Paulo, Brasil; 4 Departamento de Genética, Faculdade de Medicina de Ribeirão Preto, Universidade de São Paulo, São Paulo, Brasil; 5 Departamento de Análises Clínicas e Toxicológicas, Faculdade de Ciências Farmacêuticas, Universidade de São Paulo, São Paulo, Brasil; National Institutes of Health, United States of America

## Abstract

Limited information is available regarding the modulation of genes involved in the innate host response to *Paracoccidioides brasiliensis*, the etiologic agent of paracoccidioidomycosis. Therefore, we sought to characterize, for the first time, the transcriptional profile of murine bone marrow-derived dendritic cells (DCs) at an early stage following their initial interaction with *P. brasiliensis*. DCs connect innate and adaptive immunity by recognizing invading pathogens and determining the type of effector T-cell that mediates an immune response. Gene expression profiles were analyzed using microarray and validated using real-time RT-PCR and protein secretion studies. A total of 299 genes were differentially expressed, many of which are involved in immunity, signal transduction, transcription and apoptosis. Genes encoding the cytokines IL-12 and TNF-α, along with the chemokines CCL22, CCL27 and CXCL10, were up-regulated, suggesting that *P. brasiliensis* induces a potent proinflammatory response in DCs. In contrast, pattern recognition receptor (PRR)-encoding genes, particularly those related to Toll-like receptors, were down-regulated or unchanged. This result prompted us to evaluate the expression profiles of dectin-1 and mannose receptor, two other important fungal PRRs that were not included in the microarray target cDNA sequences. Unlike the mannose receptor, the dectin-1 receptor gene was significantly induced, suggesting that this β-glucan receptor participates in the recognition of *P. brasiliensis*. We also used a receptor inhibition assay to evaluate the roles of these receptors in coordinating the expression of several immune-related genes in DCs upon fungal exposure. Altogether, our results provide an initial characterization of early host responses to *P. brasiliensis* and a basis for better understanding the infectious process of this important neglected pathogen.

## Author Summary

Paracoccidioidomycosis is a systemic disease that has an important mortality and morbidity impact in Latin America, mainly affecting rural workers of Argentina, Colombia, Venezuela and Brazil. Upon host infection, one of the most important aspects contributing to disease outcome is the initial encounter of the *Paracoccidioides brasiliensis* fungus with dendritic cells. This phagocytic cell is specialized in decoding microbial information and triggering specific immune responses. Thus, using a molecular biology technique to examine the response of thousand of genes, we aimed to identify the ways in which murine dendritic cells interact with *P. brasiliensis* during an early time point following infection. This approach allowed us to recognize diverse modulated genes, in particular those associated with a proinflamatory response and fungal recognition. Our work provides an initial molecular characterization of early infection process and should promote further investigations into the innate host response to this important fungal pathogen.

## Introduction

The thermodimorphic fungus *Paracoccidioides brasiliensis* is the causative agent of paracoccidioidomycosis (PCM), a systemic human disease that is geographically confined to Latin America. PCM is mainly endemic in Argentina, Colombia, Venezuela and especially in Brazil, where it is the most prevalent cause of death among systemic mycoses not associated with AIDS [1].

*P.brasiliensis* infection is acquired upon the inhalation of airborne propagules derived from the saprophytic mycelium form of the fungus. Once in the lungs, *P. brasiliensis* converts to its parasitic yeast form and interacts with resident macrophages and dendritic cells (DCs) [2], [3]. DCs are the most powerful antigen-presenting cells and are uniquely able to recognize pathogen-associated molecules and activate qualitatively different adaptive T-helper (Th) cell responses [4]. Protective immunity against *P. brasiliensis* has been credited to a Th1 type response, whereas the anti-inflammatory cytokine IL-10 is generally correlated with deleterious effects in murine and human PCM [5]–[7]. Recent experiments have shown that *P. brasiliensis* infection activates DCs to migrate to regional lymph nodes and trigger a Th response [8]. The direct activation of DCs occurs via the recognition of specific microbial compounds, known as pathogen-associated molecular patterns (PAMPs), by germline-encoded pattern recognition receptors (PRRs). In particular, the Toll-like receptors (TLRs) and C-type lectin receptors (CLRs) are the most important PRRs for the recognition of fungal molecules [9], [10].

During the activation process, DCs are subject to profound changes due to the differential expression of a variety of immune-related genes, which regulate the efficiency of the DC response to pathogens [11]. From this perspective, the use of microarrays to evaluate the gene expression profiles of DCs has served as an important tool to investigate how these cells respond to infection and modulate the immune system upon interaction with different microorganisms [12]. Because little data are available about the regulation of DC genes upon *P. brasiliensis* infection, we sought to examine the transcriptional profile of murine bone marrow-derived DCs at an early time of interaction with yeast cells. Gene expression profiles were analyzed using microarray and validated using real-time RT-PCR. Cytokine secretion was also monitored. We identified 299 genes that were differentially expressed upon infection, including many genes that are involved in immunity (e.g., inflammatory cytokines, chemokines and PRRs), signal transduction, transcription and apoptosis. Additionally, we used inhibition assays to evaluate the role of the CLRs dectin-1 and mannose receptor (MR) in coordinating the expression of several immune-related genes upon exposure to *P. brasiliensis*. Taken together, our results provide a foundational description of early host gene expression changes in response to *P. brasiliensis*, which may allow a better understanding of the infectious process of this important, but neglected, fungal pathogen.

## Methods

### Ethics statement

All work was conducted with the approval of the Committee on the Ethics of Animal Experiments of the University of Sao Paulo (CEUA/FCF permit number: 2921) according to the National Council on Animal Experiments and Control (CONCEA-MCT-Brazil) guidelines.

### Mice

Male BALB/c mice were obtained from the animal laboratory of the University of São Paulo and used in experiments at 8 to 12 weeks of age. This strain has been shown to have intermediate resistance to *P. brasiliensis* infection [13].

### Fungus

The yeast form of the highly virulent *P. brasiliensis* isolate 18 was grown on Sabouraud agar and used for *in vitro* infection assays. Viability, as determined with Janus Green B vital dye (Merck), was always greater than 80%.

### Generation of bone marrow-derived DCs, *in vitro* infection and RNA extraction for microarray procedure

Bone marrow-derived DCs were generated from BALB/c mice according to the protocol described by Inaba et al. [14] with slight modifications. Briefly, mouse femurs and tibias were flushed with 2 ml phosphate buffered saline (PBS) containing 1% bovine serum albumin (BSA). Bone marrow cells were differentiated into DCs by culturing in RPMI 1640 tissue culture medium supplemented with 10% fetal calf serum (FCS), 10 mg/ml gentamicin and 50 ng/ml recombinant granulocyte-macrophage colony stimulating factor (GM-CSF) for 7 days at 37°C in a humidified atmosphere containing 5% CO_2_. On days 3 and 5, nonadherent cells (granulocytes and lymphocytes) were removed, and fresh medium supplemented with GM-CSF was added. On day 7, the non-adherent cells were removed and analyzed by flow cytometry using DC cell surface markers. Phenotypically, 80% of these cells express MHC class II, CD80, CD40, CD11b and CD11c being characterized as bone marrow-derived DCs (data not shown). Following DC generation, 10^7^ cells were infected with *P. brasiliensis* at a yeast-to-cell ratio of 1∶1 at 37°C. Using this ratio of infection, an average of 70% of DCs is engaged in phagocytosis of at least one yeast cell (data not shown). At 6 h after infection, extracellular and weakly adherent fungi were removed by washing with pre-warmed RPMI. DCs were then lysed, and total RNA was extracted with the Trizol reagent (Invitrogen) according to the manufacturer's instructions. Total RNA from control (uninfected) DCs was also extracted with Trizol.

### Microarray preparation and probe hybridization

The transcriptional response of murine DCs to infection with *P. brasiliensis* was assessed using cDNA microarrays prepared on silane-coated UltraGAPS slides (# 40015, Corning). The microarrays contained a total of 4,500 target tissue-restricted antigen cDNA sequences, representing most murine tissues and organs. The cDNA clones on the microarrays were isolated from the Soares thymus 2NbMT normalized library prepared from the thymus of C57BL/6J 4-week-old male mice, which is available at the IMAGE Consortium (http://image.hudsonalpha.org/). The microarrays were prepared based on published protocols [15] using a Generation III Array Spotter (Amersham Molecular Dynamics) according to the manufacturer's instructions and cross-linked using an ultraviolet cross-linker. The cDNA complex probes were prepared by reverse transcription using 10 µg of total DC RNA followed by labeling of the resulting cDNAs with Cy3 or Cy5 fluorochromes using the CyScribe post-labeling kit (Amersham Biosciences) and oligo dT_12–18_ as a primer. The cDNA complex probes derived from total RNA obtained from *P. brasiliensis*-infected and non-infected control DCs were labeled with Cy5 using the CyScribe post-labeling kit (GE Healthcare). As a control for the hybridization procedure, we used equimolar quantities of Cy3-labeled cDNA generated from total RNA isolated from different mouse organs (thymus, spleen and lung). This approach allowed for the amount of cDNA targeted in each microarray spot to be estimated. The 15-h period of hybridization, followed by washing, was performed in an automatic slide processor system (ASP; Amersham Biosciences), and the microarrays were scanned with a Generation III laser scanner (Amersham Biosciences).

### Microarray data analysis

Microarray image quantification was performed using Spotfinder (http://www.tm4.org/spot.nder.html). The normalization process was carried out using the R platform (http://www.r-project.org), and statistical analyses were conducted with Multiexperiment Viewer (MeV) (version 3.1; http://www.tm4.org/mev.html). After normalization, SAM (Significance Analysis of Microarrays) was used to identify statistically significant differences in gene expression between the experimental and control conditions [16]. SAM computes a statistic for each gene, measuring the strength of the relationship between gene expression and the response variable (*P. brasiliensis* infected and non-infected DCs groups). It uses repeated permutations of the data to determine if the expression of any genes is significantly related to the response. The threshold for significance is determined by a tuning parameter delta based on the false-positive rate or false discovery rate (FDR). A high stringent FDR 0.5% (Delta = 1.017) and *q*-value≤0.05 were selected. The *q*-value (*p*-value adapted to multiple-testing conditions) for each gene is the lowest FDR at which that gene modulation is called significant. SAM analysis also provides the optional fold change parameter, to ensure that significant modulated genes change by at least a pre-specified amount. In the present work, a fold-change cutoff set to 1.2, simulating 20% cutoff, was used. Microarray data were deposited according to MIAME (Minimum Information About a Microarray Experiment) guidelines in the ArrayExpress databank (http://www.ebi.ac.uk/arrayexpress/) under accession number A-MEXP-2009.

### Mannose and dectin-1 receptor inhibition assays and cytokine quantitation

Bone marrow-derived DCs, generated as described above, were plated at a concentration of 1×10^6^ cells/ml in 24-well cell-culture-treated plates and pre-incubated for 30 min at 37°C with 100 µg/ml α-mannan obtained from *Saccharomyces cerevisae* (Sigma-Aldrich) or 200 µg/ml laminarin (Sigma-Aldrich) to block the mannose or dectin-1 receptors, respectively. The DCs were then infected with *P. brasiliensis* yeast cells, following the protocol for the *in vitro* infection assay described above for the microarray experiments. IL-12, IL-10 and TNF-α secretion were measured from culture supernatants using commercially available ELISAs according to the manufacturer's recommendations (BD Pharmingen).

### Quantitative RT-PCR (qRT-PCR)

qRT-PCR was used to validate the differential modulation of DC genes revealed by the microarray experiment and for the analysis of DC gene expression following mannose and dectin-1 receptor inhibition. To remove any genomic DNA contamination, total RNA extracted from cells from both experimental conditions was treated with RNase-free DNaseI (Promega) and precipitated with ethanol. These DNA-free RNA samples were then used for qRT-PCR. Equal amounts of RNA (1 µg) were reverse transcribed (Superscript III, Invitrogen) using an oligo(dT)_12–18_ primer and submitted to real time PCR. Amplification assays were carried out on a 7500 Fast Real-Time PCR System with SDS software (Applied Biosystems) in 10 µl reactions containing 0.2 µM of each primer, 5 µl SYBR Green PCR master mix (2×) and 0.2 µl cDNA. After initial denaturation at 95°C for 20 s, amplifications were carried out for 40 cycles at 95°C for 3 s and 60°C for 20 s. To confirm the amplification specificity, the PCR products were subjected to a melting curve analysis. The comparative C_T_ (crossing threshold) method [17], using the constitutively expressed murine 40S ribosomal protein S9 (*RPS9*) gene as a control, was used to evaluate the expression (fold change) of each gene of interest. All primers used for qRT-PCR (Table S1) were based on sequences obtained from the mouse transcriptome database (http://www.informatics.jax.org) and designed with Primer3, which is available online (http://www-genome.wi.mit.edu).

### Statistical analyses

GraphPad Prism 5.0 (GraphPad Software) was used for statistical analyses. The paired two-tailed Student's t test was used, and a P value≤0.05 was considered significant. In addition, multiple group comparisons were conducted by one-way ANOVA followed by Bonferroni tests, as appropriate.

## Results

### Transcriptional response of DCs upon infection with *P. brasiliensis* and validation of microarray data

The pattern of gene expression in murine bone marrow-derived DCs infected with *P. brasiliensis* yeast cells was assessed using microarray. Previous studies have shown that a 4- to 6-h co-cultivation of *P. brasiliensis* yeast cells with murine bone marrow-derived DCs stimulated significant phagocytosis [18]. Based on these results, we selected a 6-h infection period because it represented an early time point of fungal internalization by DCs. For each condition (*P. brasiliensis*-infected or non-infected control DCs), two independent cDNA microarray experiments were performed with 4,500 cDNA clones on each microarray. The analysis of DC gene-expression data using SAM revealed significant changes in the expression profiles of 299 genes (81 up-regulated and 218 down-regulated) in response to infection with *P. brasiliensis* (Table S2). Based on the findings of previous fungal-phagocyte interaction studies, we selected modulated genes and clustered them into different functional categories shown in Tables 1 and 2.

**Table 1:** Selected genes up-regulated in murine dendritic cells after 6 h of infection with *P. brasiliensis*.

Category	Description	Gene	Clone ID*	Fold Change
**Immune Response**	Tumor necrosis factor alpha	*TNF-α*	5213134	1.91
	Interleukin 12b	*IL12B*	750641	1.32
	Chemokine (C-X-C motif) ligand 10	*CXCL10*	5327290	1.45
	Chemokine (C-C motif) ligand 22	*CCL22*	577486	2.94
	Chemokine (C-C motif) ligand 27	*CCL27*	1446118	1.34
	CD27 antigen	*CD27*	583084	1.42
	CD53 antigen	*CD53*	576862	1.38
	Complement component 1	*C1QB*	583277	1.28
	Interferon-activated gene 203	*IFI203*	582096	2.27
	Immunoresponsive gene 1	*IRG1*	577102	1.43
	Guanylate -binding protein 2	*GBP2*	583808	1.73
	Lymphocyte antigen 86 (MD1)	*LY86*	583305	1.22
**Signal Transduction**	Toll-interacting protein	*TOLLIP*	4951445	2.23
	Diacylglycerol kinase gamma	*DGKG*	576044	1.37
	Nuclear receptor subfamily 3, group C, member 1	*NR3C1*	576681	1.96
**Transcription**	Protein inhibitor of activated STAT1	*PIAS1*	577047	1.6
	P105 subunit of nuclear factor kappa B	*NFκB*	575033	1.42
	Zinc finger homeobox 1a	*ZFHX1*	583581	3.15
	TAF4A RNA polymerase II, (TBP)-associated factor	*TAF4A*	640087	3.50
**Apoptosis**	Caspase 2	*CASP2*	573760	1.43
	BCL-2-associated transcription factor 1	*BCLAF1*	641008	1.28
	Death effector domain-containing	*DEDD*	576731	1.48

*cDNA clones were obtained from the Soares mouse thymus 2NbMT normalized library, available from the IMAGE Consortium (http://image.hudsonalpha.org/).

**Table 2:** Selected genes down-regulated in murine dendritic cells after 6 h of infection with *P. brasiliensis*.

Category	Description	Gene	Clone ID*	Fold Change
**Immune response**				
	Chemokine (C-C motif) ligand 25	*CCL25*	575567	−1.63
	CD63 antigen	*CD63*	574568	−1.33
	CD209a antigen (DC-SIGN)	*CD209A*	3413503	−1.37
	Toll-like receptor 4	*TLR4*	6047339	−1.26
	Fc receptor, IgG, high affinity I	*FCGR1*	575540	−1.33
	Integrin, alpha 6	*ITGA6*	44814	−1.63
	Integrin beta 2 (CD18)	*ITGB2*	583119	−1.51
	Integrin alpha M (CD11b)	*ITGAM*	3966116	−3.03
**Signal transduction**				
	Mitogen-activated protein kinase 6	*MAPK6*	50506	−1.61
	Mitogen-activated protein kinase 3	*MAPK3*	641003	−1.63
	Mitogen-activated protein kinase kinase kinase 10	*MAP3K10*	639683	−1.40
**Transcription**	NFκB repressing factor	*NκRF*	21961	−1.33
	Immediate early response 5	*IER5*	639672	−1.47
	Signal transducer and activator of transcription 2	*STAT2*	5351573	−1.63
	Signal transducer and activator of transcription 6	*STAT6*	5349719	−1.40
	Interleukin enhancer-binding factor 2	*ILF2*	1264669	−1.63
**Chaperone**	Heat shock protein 8	*HSPA8*	575712	−1.69
	Heat shock protein 90 kDa beta (Grp94), member 1	*HSP90B1*	575854	−1.36
**Apoptosis**				
	CASP8 and FADD-like apoptosis regulator	*CFLAR*	640005	−1.75
	Reticulon 4	*RTN4*	640358	−1.47

*cDNA clones were obtained from the Soares mouse thymus 2NbMT normalized library, available at the IMAGE Consortium (http://image.hudsonalpha.org/).

**Figure 1:**
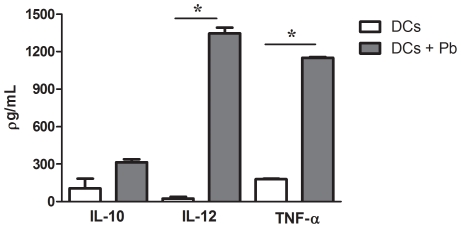
Quantification of cytokine secretion by murine dendritic cells infected with *P. brasiliensis*. BALB/c bone marrow-derived DCs were infected with live *P. brasiliensis* (Pb) yeast cells (1∶1 ratio of yeast to DCs). Culture supernatants were harvested after 6 h, and secreted protein levels were measured using ELISA. Data are reported as the mean ± standard deviation. * P<0.05.

Genes encoding cytokines such as tumor necrosis factor alpha (TNF-α) and interleukin 12 (IL-12) were up-regulated. The induction of *TNF-α* was confirmed by qRT-PCR (Table 3). In addition, protein levels, as assayed by ELISA, were increased, consistent with the increased accumulation of *TNF-α* and *IL-12* mRNA in DCs exposed to *P. brasiliensis*. In contrast, no significant IL-10 secretion was observed (Figure 1). *P. brasiliensis* infection also modulated the expression of genes encoding chemokines, which are critical chemotactic factors in the immune system. As shown in Tables 1 and 2, the genes encoding the chemokines CCL22, CCL27 and CXCL10 were up-regulated, whereas *CCL25* transcription was decreased. Moreover, a ten-fold increase in *CCL22* transcript levels was observed using qRT-PCR, confirming the microarray data (Table 3).

**Table 3:** Real-time PCR validation of microarray data.

Clone ID	Gene	Fold Change	
		Microarray6 h	qRT-PCR*6 h
577486	*CCL22*	2.94	10.28±1.32
5213134	*TNF-α*	1.91	19.42±0.98
575033	*NFκB*	1.42	4.42±0.37
21961	*NκRF*	−1.33	−1.82±0.02
6047339	*TLR4*	−1.26	−1.82±0.07
4753177	*TLR2*	NM	1.12±0.06
190887	*MYD88*	NM	1.06±0.06

*Fold-change values were determined after normalization to *Rps9* using the comparative threshold method. Values indicate mean fold change ± SD of two independent experiments.

NM: not significantly modulated, as shown by microarray.

The expression levels of some DC membrane receptor genes that are associated with immune responses were also significantly modulated. Microarray results revealed that the genes encoding the CLR receptor DC-SIGN (CD209a), the IgG receptor FcγR1 and TLR4 were down-regulated at an early time point after yeast infection, with fold-change values (FC) of −1.37, −1.33 and −1.26, respectively (Table 2). Although the decrease in *TLR4* mRNA levels at 6 h after infection was confirmed by qRT-PCR (Table 3), no significant modulation was found at a later time point, 24 h after infection (data not shown). Microarray and qRT-PCR data showed that *TLR2* gene expression did not appear to be influenced by the presence of fungal cells at 6 h (Table 3). Likewise, the expression levels of two other TLRs family members (TLR6 and TLR9, data not shown) and the universal adaptor molecule of the TLR signaling pathway, MyD88, were not modulated. The unchanged expression of MyD88 was also validated using qRT-PCR (Table 3). In this manner, genes that encode negative regulators of TLR-mediated signaling, such as TOLLIP (Toll-interacting protein) and the lymphocyte antigen 86 (LY86) known as MD1, were up-regulated 6 h after infection (Table 1).

Integrins are a family of proteins whose members are involved in a variety of cell-matrix and cell-cell adhesion processes and signaling events that are central to immunologic and inflammatory processes [19]. These proteins are heterodimeric transmembrane glycoproteins that consist of a series of related α and β subunits. As shown in Table 2, down-regulation of the DC integrin genes *ITGAM* (CD11b), *Itg2b* (CD18) and *ITGA6* was observed in response to *P. brasiliensis* infection. The α subunit of CD11b bound to the β subunit CD18 is known as integrin CD11b/CD18 or Complement Receptor 3 (CR3). In addition to its ability to promote the phagocytosis of iC3b-opsonised particles, CR3 recognizes exogenous ligands, such as β-glucan, and has been implicated in DC responses to fungi [10], [20].

Infected DCs also showed altered expression of interferon-inducible genes and genes encoding transcription factors. *IRG1*, or immunoresponsive gene 1, was up-regulated in response to *P. brasiliensis* infection (Table 1). Interestingly, Degrandi et al. [21] showed that the mRNA level of this gene increased following TNF-α and IFN-γ treatment. Another interferon-inducible gene, *IFI203*, was shown to be up-regulated, with an FC of 2.27. As shown in Tables 1 and 3, after 6 h of *in vitro* infection with *P. brasiliensis* yeast cells, *NFκB* was up-regulated in DCs. Its product is the major transcription factor that induces the expression of pro-inflammatory genes. Moreover, *NκRF* was down-regulated. *NκRF* encodes a transcriptional repressor that binds to specific negative regulatory elements (NREs) to counteract NFκB activity at certain gene promoters [22]. Other down-regulated genes involved in transcriptional regulation included *STAT2*, *STAT6*, *IER5* and *ILF2* (Table 2).

Another important group of genes with altered expression consisted of those related to apoptosis. The pro-apoptotic genes *CASP2*, *BCLAF1* and *DEDD* were up-regulated (Table 1), while *BTG2* and *RTN4* were down-regulated (Table 2). Moreover, the expression of the anti-apoptotic gene *CFLAR* was inhibited, with an FC of 1.75 (Table 2).

### The expression of dectin-1 and MR in DCs infected with *P. brasiliensis* and receptor inhibition assays

**Figure 2:**
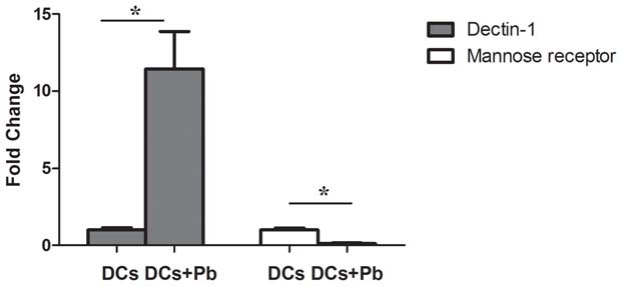
Relative quantification of dectin-1 and mannose receptor transcripts in dendritic cells infected with *P. brasiliensis*. BALB/c bone marrow-derived DCs were cultured for 6 h with or without *P. brasiliensis* yeast cells (Pb). Then total RNA was extracted from the DCs and used in qRT-PCR assays. Fold change values were determined after each gene was normalized to the constitutively expressed *RPS9* gene using the comparative threshold method. Data are reported as the mean ± standard deviation. * P<0.05.

**Figure 3:**
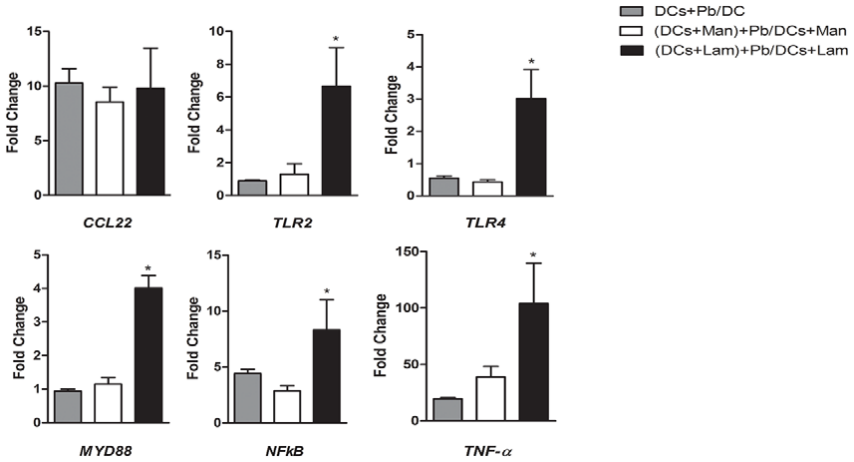
Effect of mannan and laminarin on the accumulation of selected immune-related transcripts in dendritic cells infected with *P. brasiliensis*. Murine bone marrow-derived DCs were incubated with mannan (Man) or laminarin (Lam) at 100 and 200 µg/ml, respectively, for 30 min. Subsequently, *P. brasiliensis* yeast cells (Pb) were added to DCs at a ratio of 1∶1, and the co-culture was incubated for 6 h. Total RNA was extracted from DCs and used in qRT-PCR assays. Fold-change values were determined after each gene was normalized to *RPS9* using the comparative threshold method. Data are reported as the mean ± standard deviation. * P<0.05 compared to the DCs+Pb/DCs group.

As described above, the expression of several DC receptor-encoding genes, including opsonin-dependent receptors (CR3 and FcγR1) and non-opsonin dependent PRRs (TLR2, TLR4, TLR6, TLR9 and DC-SIGN), were analyzed using microarray. The genes encoding TLR4, CR3, FcγR1 and DC-SIGN were down-regulated, while TLR2, TLR6 and TLR9 were not differentially regulated. To obtain a more comprehensive analysis of immune receptor expression, we evaluated the expression profiles of dectin-1 and MR, two major fungal CLRs [23] that were not included in the microarray target cDNA library. qRT-PCR analysis revealed that the dectin-1 receptor gene was up-regulated by more than ten fold after 6 h of *P. brasiliensis* infection, while MR was down-regulated (Figure 2). These results prompted us to investigate the possible effects of dectin-1 and MR on the transcriptional profiles of six immune-related genes (*CCL22*, *TNF-α*, *NFκB*, *TLR2*, *TLR4* and *MYD88*) that were selected based on the microarray results and importance to the fungi-host interaction. DCs were incubated with laminarin or mannan to block dectin-1 and MR, respectively. After 30 minutes, DCs were infected with *P. brasiliensis* for 6 h, total RNA was extracted, and the expression profiles of the selected immune-related genes were determined by qRT-PCR. The comparative C_T_ method [17], using the constitutively expressed murine *RPS9* gene as a control, was used to evaluate the expression (fold change) of each interest gene. In this analysis we have considered each gene expression ratio obtained between *P. brasiliensis* infected and uninfected DCs with respect to host cells treatment or not with mannan or laminarin. In the presence of mannan, no differences in transcript accumulation were observed. In contrast, DCs treated with laminarin before infection showed a significant up-regulation of all six genes investigated, except *CCL22* (Figure 3). These data suggest that dectin-1 plays a prominent role in the coordination of gene expression during the initial phase of *P. brasiliensis* infection.

### The effects of mannan and laminarin on cytokine secretion by murine DCs infected with *P. brasiliensis*

**Figure 4:**
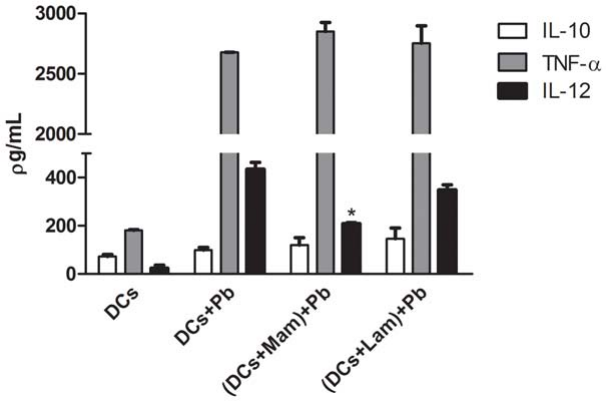
Effect of mannan and laminarin on cytokine secretion by murine dendritic cells infected with *P. brasiliensis*. Murine bone marrow-derived DCs were incubated with mannan (Man) or laminarin (Lam) at 100 and 200 µg/ml, respectively, for 30 min. Subsequently, *P. brasiliensis* yeast cells (Pb) were added to DCs at a ratio of 1∶1, and the co-culture was incubated for 6 h. Culture supernatants were harvested and secreted protein levels were measured using ELISA. Data are reported as the mean ± standard deviation. * P<0.05 compared to the DCs+Pb group.

The secretion of IL-10, IL-12 and TNF-α was evaluated after inhibitor assays (Figure 4). Treatment with laminarin prior to infection did not alter the secretion of any of these three cytokines when DCs were exposed to *P. brasiliensis*, whereas MR inhibition by mannan significantly reduced IL-12 secretion. Treatment with laminarin or mannan alone (i.e., without subsequent infection) had no effect on basal the secretion of any of these three cytokines by DCs (data not shown).

## Discussion

To our knowledge, this is the first study that has investigated the gene expression profile of mouse DCs in response to a primary pathogenic fungus. Previous studies have evaluated gene expression in human monocyte- and mouse splenic-derived DCs in response to the opportunistic fungi *Candida albicans* and *Cryptococcus neoformans*, respectively [12], [24]. Our microarray data identified several genes whose expression is modulated at an early time point after bone marrow-derived DCs are exposed to *P. brasiliensis* yeast cells. In particular, genes related to immune responses (mainly inflammation-associated genes), signal transduction, transcriptional regulation and apoptosis were altered.

DCs connect innate and adaptive immunity by recognizing pathogen-associated molecules and producing cytokines that subsequently drive qualitatively different adaptive Th responses. IL-12 produced by DCs is the key cytokine that stimulates a Th1-type cell-mediated response, the major source of immunity against systemic fungal infections [10]. Here, we demonstrate the increased production of IL-12 mRNA and protein by BALB/c DCs infected with *P. brasiliensis* for 6 hours. Considering that this mouse lineage has intermediate resistance to *P. brasiliensis* infection [13], we could draw a parallel with previous observations demonstrating that DCs derived from resistant mice (A/J) stimulate a Th1 response *in vitro* more efficiently than DCs derived from susceptible mice (B10.A) when pulsed with the immunodominant *P. brasiliensis* antigen, gp43 [25]. Diverse antigen-recognition and -processing mechanisms in DCs from resistant and susceptible mice, which would give rise to differential production of IL-12, could be involved in determining susceptibility to this fungus. In fact, gp43-pulsed DCs from resistant mice were later reported to secrete higher levels of IL-12 than those from susceptible mice, but this increase was not statistically significant [26]. Based on this observation, the authors speculated that IL-12 is not the key factor for promoting a Th1-type response in A/J mice. Other mechanisms, such as the high expression of the costimulatory molecule CD80, concomitant with low production of IL-4 and IL-6, could contribute to the induction of Th1 cells. In contrast to these results, a recent study described a role for IL-12 in determining resistance to experimental PCM. Mice that are deficient for the IL-12p40 subunit produce no detectable IFN-γ and high levels of IL-10 protein, and this phenotype is associated with uncontrolled fungal proliferation and dissemination [27]. Moreover, Moraes-Vasconcellos et al. [28] described a patient with disseminated PCM who harbored a primary immunodeficiency in the beta 1 subunit of the IL-12/IL-23 receptor.

Our microarray analysis showed that, in addition to *IL-12*, other proinflammatory cytokine- and chemokine-encoding genes (*TNF-α*, *CCL22*, *CCL27* and *CXCL10*) were induced by *P. brasiliensis* exposure. Interestingly, microarray studies published by Lupo et al. [24] demonstrated the up-regulation of all these genes (except *CCL27*) in murine DCs exposed to acapsular *C. neoformans* relative to DCs exposed to encapsulated strains. These results are consistent with the role of the polysaccharide capsule as the main virulence factor for this important opportunistic fungus and the ability of the capsule to act as a shield from immune recognition and activation. Notably, *IL-12, CCL22* and *CXCL10* are part of a cluster of murine DCs signature expressed genes that discriminate very accurately between inflammatory and non-inflammatory stimuli, such as lipopolysaccharide and dexamethasone, respectively [29]. Another signature inflammatory DC gene identified in our study was *NFκB1*, which encodes the p105 subunit of the NFκB protein. NFκB is a master regulator of gene transcription during development and inflammatory processes and plays a critical role in the activation of innate and adaptive immunity [30]. Among the known targets of NFκB, we were able to show that DCs up-regulated *IL-12, TNF-α, CCL22 and CXCL10* in response to *P. brasiliensis*. It is important to note that levels of *NκRF* (a nuclear inhibitor of NFκB activity) mRNA were reduced concomitant with *NFκB* induction.

TNF-α is a cytokine that is critical for the successful control of fungal infections and the development of a Th1-dependent response. This cytokine augments the cytotoxic activity of activated macrophages, induces chemokine production and, along with IFN-γ, regulates granuloma formation [31]. *P. brasiliensis*-infected mice lacking the p55 subunit of TNF-α receptor were reported to develop severe PCM associated with non-organized granulomas [32]. In experimental pulmonary cryptococcosis, the transient depletion of TNF-α production during the early innate immune response permanently impaired the long-term control of fungal growth. This effect was coupled with a temporary decrease in neutrophil lung influx, reduced IL-12 production and recruitment of DCs to draining lymph nodes [33]. In this context, the early *TNF-α* gene expression and protein production observed in the current study is likely to be associated with the induction of a protective response. As discussed above, and consistent with a proinflammatory scenario, we observed the up-regulation of several chemokines (CCL22, CXCL10 and CCL27) in our study. Chemokines play a major role in mediating the extravasation and accumulation of specific leukocytes at sites of infection, which is crucial for the local control of fungal invasion [34]. Both CXCL10 and CCL22 are mainly chemotactic for monocytes and T-lymphocytes. CCL22 is also chemotactic for DCs, the major cell source for this chemokine *in vivo* and *in vitro* [35]. Notably, our group has previously shown the induction of *CCL22* gene, among other chemokine-encoding genes, in peritoneal murine macrophages that were infected with *P. brasiliensis* [36]. In addition to its role as a chemoattractant, CCL22 enhances the microbicidal activity of macrophages by stimulating a strong respiratory burst and lysosomal enzyme release [37]. Similarly, CXCL10 not only induces leukocyte migration but also up-regulates the production of Th1 cytokines (mainly IFN-γ) and down-regulates the production of Th2 cytokines upon interaction with its receptor on T- cells [38]. In humans, single nucleotide polymorphisms in the *CXCL10* gene lead to reduced chemokine production by DCs exposed to *Aspergillus fumigatus*, causing invasive aspergillosis [39].

Of particular interest in our study was the assessment of transcriptional modulation of genes encoding PRRs, which specifically interact with pathogen PAMPs and thus regulate the production of various immune-related molecules. A great number of PRRs have been identified; of these, the TLRs and CLRs families are of major interest because they appear to have critical roles in fungal immunity [40]–[41]. Regarding TLRs, we observed no appreciable modulation of the expression of *TLR2*, whereas *TLR4* was down-regulated. In accordance, the expression of *MYD88* was unaltered in *P. brasiliensis*-infected DCs, as shown using microarray and qRT-PCR. MYD88 is a universal adaptor molecule in the TLR signaling pathway that ultimately activates NFκB and thus affects subsequent cytokine and chemokine production [42]. Interestingly, the importance of MYD88 signaling in the experimental murine model of PCM is controversial. Gonzalez et al [43] showed that this adaptor protein is not necessary for the effective control of blood-borne disseminated *P. brasiliensis* infection. In contrast, a recent study demonstrated that MyD88-dependent signaling participates in the induction of protective immune host defense against pulmonary PCM [44]. Different fungal strains and routes of infection may have been responsible for this divergence. In our study model, the limited participation of TLR-mediated signal transduction in response to *P. brasiliensis* is further supported by the fact that two genes (*TOLLIP* and *MD1*) that encode negative regulators of this signaling pathway were induced. TOLLIP is an adaptor molecule that can associate with TLR2 and TLR4 to inhibit MyD88 binding and activation [45]. Indeed, the overexpression of TOLLIP precludes NFκB activation in response to TLR2 and TLR4 agonists [46]. Likewise, MD1 is a helper molecule for RP105, a TLR homolog that acts as a physiological negative regulator of TLR4 responses [47]. These results suggest that TLRs may have only a minor role in the host responses elicited by *P. brasiliensis*. In fact, TLR2 and TLR4 deficient mice infected with this fungus demonstrated equivalent mortality rates compared with wild-type littermates [48]–[49]. Interestingly, similar results were obtained in TLR2 and TLR4 knockout mice infected with *C. neoformans* [50].

Our microarray data suggesting limited role for TLR-mediated signaling in response to *P. brasiliensis*, coupled with significant production of IL-12 and TNF-α but not IL-10, prompted us to search for a TLR2-4/MyD88-independent mechanism that could explain the induction of these proinflammatory cytokines in DCs. In accordance, bone marrow-derived DCs from mice deficient in the *TLR2* and *TLR4* genes and infected with *C. neoformans* had no significant reduction in IL-12 and TNF-α protein levels [50]. Because receptors of the C-type lectin family, particularly dectin-1 and MR, have been reported to be critical for the recognition of fungi and the activation of macrophages and DCs [40], we sought to evaluate their expression using qRT-PCR analysis (these genes were not represented in our microarray). The dectin-1 receptor gene, whose product recognizes β-(1,3)-glucans on the cell walls of fungi, was up-regulated by ten fold, whereas the MR gene transcription was diminished. These results suggest that dectin-1 participates in the induction of TNF-α and the production of IL-12. Unexpectedly, the blockade of this receptor with laminarin did not significantly reduce the production of these cytokines by DCs. This apparent contradiction may be because dectin-1 inhibition results in significant induction of the *TLR2*, *TLR4*, *MYD88*, *NFκB*, and *TNF-α* genes, which could be involved in the sustained IL-12 and TNF-α production, probably via TLR-mediated signaling. Thus, dectin-1 may act as negative regulator of the TLR signaling pathway in our model. These results could be associated with the intermediate PCM resistance pattern of the mouse strain used in our study. DCs from mice that are susceptible to this fungus, but not resistant mice, secrete significant amounts of IL-10 in a TLR2/dectin-1 collaborative signaling-dependent manner. Furthermore, *TLR2* gene expression was only induced by *P. brasiliensis* in susceptible mice and its deletion suppress IL-10 production [51]. Altogether, these results suggest that dectin-1 signaling down-regulates the expression of TLR-associated genes, leading to a Th1-like response with little anti-inflammatory IL-10 production (i.e., no collaborative dectin-1 and TLR signaling). MR blockade did not alter TNF-α or IL-10 secretion, as observed in DCs treated with laminarin. However, despite the downregulation of the MR-encoding gene, a significant reduction in IL-12 expression was observed after treatment with mannan. MR recognizes *P. brasiliensis*, *C. neoformans* and *C. albicans*, and this receptor has been implicated in mediating the production of pro-inflammatory cytokines, including IL-6, TNF-α, IL-1β and IL-12 [40]. These data could indicate a lack of correlation between the MR mRNA level and the expression of the functional receptor. Alternatively, because we used fungal mannan in our inhibition assay and this mannose polymer is also recognized by other PRRs, such as DC-SIGN, SCARF1 and mincle, we could not rule out the possibility that one or more of those receptors promote the induction of IL-12 secretion.

In summary, our findings demonstrate that *P. brasiliensis* triggers the accumulation of mRNAs for genes that encode proinflammatory cytokines and chemokines as well as other molecules involved in the early response of DCs to this fungus. These results provide a better understanding of the molecular pathogenesis of PCM and should promote future investigations into the innate host response to this fungus, including *in vivo* analysis. Of particular interest were the results regarding receptor inhibitor assay, because the mechanisms by which dectin-1 negatively regulates TLR-associated gene expression remain to be determined.

The information concerning genes and proteins accession numbers mentioned in the manuscript (text and Tables 1 and 2) is described below, following the criteria: **Gene name - Gene Accession number/Protein Accession number** Il12b (Il12p40) - NM_008352/NP_032378; Cxcl10 - AK146144/NP_067249; Ccl22 - AF052505/NP_033163; Ccl27a - AK146066/NP_035466; Cd27 - L24495/NP_001036029; Cd53 - NM_007651/NP_031677; C1qb - AK152764/NP_033907; Ifi203 - AK172243/NP_032354; Irg1 - NM_008392/NP_032418; Gbp2 - BC032882/NP_034390; Ly86 - AK172197/NP_034875; Tollip - BC062139/NP_076253; Nr3c1 - NM_008173/NP_032199; Pias1 - AK075708/NP_062637; Nfkb1 - AK036827/NP_032715; Taf4a - NM_001081092/NP_001074561; Casp2 - BC034262/NP_031636; Ccl25 - AK154211/NP_033164; Cd209a - AY049062/NP_573501; Fcgr1 - AK033874/NP_034316; Itgb2 - AK136502/NP_032430; Itgam - NM_001082960/NP_032427; Mapk3 - BF579077/NP_036082; Map3k10 - NM_001081292/NP_001074761; Ier5 - NM_010500/NP_034630; Stat2 - AF206162/NP_064347; Stat6 - NM_009284/NP_033310; Otud7b - BC141397/NP_001020785; Hspa8 - BC006722/NP_112442; Hsp90b1 - AK160827/NP_035761; Nadk - NM_001159637/NP_619612; Rtn4 - NM_194054/Q99P72.

## Supporting Information

Table S1 **Oligonucleotides used for qRT-PCR analysis.**
(DOC)

Table S2 **Complete list of probe sets identified by SAM as being differentially modulated in DCs infected with** ***P. brasiliensis*** **.**
(XLS)
