# Supplementary material for: Murine Dendritic Cells Transcriptional Modulation upon Paracoccidioides brasiliensis Infection
Source: PLoS Negl Trop Dis. 2012 Jan 3;6(1):e1459. doi: 10.1371/journal.pntd.0001459 (PMC3250510; doi:10.1371/journal.pntd.0001459)
Supplement: Table S1 — Oligonucleotides used for qRT-PCR analysis. (DOC) [file pntd.0001459.s001.doc]

Table S1. **Oligonucleotides employed in Real time RT-PCR analysis.**

| **Gene** | **Forward primer (5’→ 3’)** | **Reverse primer (5’→ 3’)** |
| --- | --- | --- |
| *RPS9* | CGCCAGAAGCTGGGTTTGT | CGAGACGCGACTTCTCGAA |
| *CCL22* | GACCTCTGATGCAGGTCCCTAT | GGATGTAGTCCTGGCAGCAGATA |
| *NFκB* | AGCCAGCTTCCGTGTTTGTT | AGGGTTTCGGTTCACTAGTTTCC |
| *NκRF* | ACCTTTCAACCTACGATGGTCAGA | GAGCTCTCACATGGAATTTGGAA |
| *TNF-α* | GTACCTTGTCTACTCCCAGGTTCTCT | GTGGGTGAGGAGCACGTAGTC |
| *TLR2* | aagaggaagcccaagaaagc | cgatggaatcgatgatgttg |
| *TLR4* | gaacaaaactctggggcctaaac | catgggctctcggtccatag |
| *MYD88* | actggcctgagcaactagga | cgtgccactacctgtagcaa |
| *Dectin1* | TAATCTCTGCCCCCAAAACC | AACTGCTTCGACCCAGACCT |
| *Mannose receptor* | TCTTGGGTCGGATGATTCTG | GCCTGCTCTTCCTCTGACCT |
